# Association of lymphocyte count and serum albumin concentration with telomere length in Chinese sanitation workers

**DOI:** 10.1371/journal.pone.0311736

**Published:** 2024-10-10

**Authors:** Xingxu Song, Dafeng Lin, Dianpeng Wang, Shaofan Weng, Shuyi Qiu, Wei Zhou, Aipin Xiao, Naixing Zhang

**Affiliations:** 1 Public Health Department, Jilin University, Changchun, China; 2 Occupational Health Department, Shenzhen Prevention and Treatment Center for Occupational Diseases, Shenzhen, China; 3 Medical Laboratory, Shenzhen Prevention and Treatment Center for Occupational Diseases, Shenzhen, China; 4 Public Health and Preventive Medicine Department, Shantou University, Shantou, China; 5 Public Health Department, Southern Medical University, Guangzhou, China; Rutgers: Rutgers The State University of New Jersey, UNITED STATES OF AMERICA

## Abstract

**Objective:**

This study aimed to examine the association between inflammation-related indicators (IRIs) and telomere length (TL) in Chinese sanitation workers.

**Methods:**

This study adopted a case-control design, conducted from January to December 2022 in Shenzhen, a city in eastern China. A total of 80 sanitation workers, as well as 80 matched controls, were randomly recruited from the Luohu district of Shenzhen city in China. Their blood samples were collected and analyzed for the IRIs and TL in the Medical Laboratory of Shenzhen Prevention and Treatment Center for Occupational Diseases. The relationship between IRIs and TL was analyzed using multivariate linear regression, and their dose-response relationship was explored using restricted cubic spline analysis.

**Results:**

The systemic inflammatory index (SII), platelet-to-lymphocyte ratio (PLR), and neutrophil-to-lymphocyte ratio (NLR) were significantly elevated in the sanitation workers in comparison to the controls. Moreover, the lymphocyte count (LYM), serum albumin concentration (ALB), and TL were found to be lower in the sanitation workers compared to the controls (*P* < 0.05). After adjusting for potential confounding variables, LYM was negatively correlated with TL in the sanitation workers (β = -0.31, 95% CI: -0.57, -0.05), whereas no correlation was observed in the controls. Furthermore, ALB demonstrated a non-linear relationship with TL in sanitation workers.

**Conclusion:**

We found higher novel inflammatory markers (SII, PLR, and NLR) in the sanitation workers, and identified a correlation between LYM and ALB with shortened TL in them, providing new evidence for the effect of elevated inflammation on accelerated aging in Chinese sanitation workers.

## Introduction

Sanitation workers play a pivotal role in the provision and maintenance of sanitation systems, which are essential for maintaining safe sanitation services and protecting public health in urban environments [1]. The work of sanitation workers is frequently conducted in an environment that is potentially harmful to health. This environment is characterized by exposure to dust, fumes, odors, hazardous substances and chemicals, and dangerous machinery [2]. In numerous developing countries, sanitation workers are more susceptible to ad hoc or unenforced environmental and labor protections, as well as a deficiency of occupational health and safety measures [3]. Several studies have demonstrated that sanitation workers are at an increased risk of developing respiratory infections or other adverse health conditions [4–6]. The awkward postures and heavy lifting that are common among sanitation workers increase the likelihood of developing musculoskeletal disorders [7–9]. Furthermore, the exposure of sanitation workers to environmentally hazardous substances and the performance of sewage treatment work may be associated with increased systemic inflammation and inflammatory markers [10–12]. A recent review has confirmed the elevated risk of poorer health among sanitation workers [13]. Consequently, it is imperative to comprehend the health status of sanitation workers and the health risks they confront, to inform the formulation of policies designed to safeguard their well-being, particularly in developing countries.

Telomere length (TL) considered as a biomarker of biological aging [14, 15]. Shortened TL is linked to a shorter lifespan and an increased risk of age-related diseases, including cancer, cardiovascular disease, type 2 diabetes, and all causes of mortality [16–19]. In addition to age, inflammation is another key factor contributing to TL shortening [20]. The systemic inflammatory index (SII), platelet-to-lymphocyte ratio (PLR) and neutrophil-to-lymphocyte ratio (NLR) are recently proposed inflammatory factors based on the complete blood count, which can be reliable indicators of the immune and inflammatory status of the body [21]. Additionally, low lymphocyte count (LLC) is a common laboratory finding that is indicative of an acute inflammatory response [22]. One study reported that LLC was significantly associated with frailty [23]. Aging is associated with an increase in lymphocyte apoptosis [24], and LLC has become a prognostic indicator in patients with chronic or acute heart failure [25]. Conversely, serum albumin concentration (ALB) has been associated with nutrition and inflammation [26]. A reduction in ALB is strongly correlated with the aging process, reflecting a multitude of pathological conditions, including inflammation, frailty, and a variety of other pathological conditions, such as cancer, rheumatoid arthritis, and abnormal liver function [26, 27]. Furthermore, low ALB may be associated with an increased risk of developing sarcopenia [28].

This study sought to examine the relationship between IRIs and TL in sanitation workers to help inform the development of strategies for the management and protection of sanitation workers’ health especially in developing countries such as China. A random sample of 80 sanitation workers and 80 comparable controls were recruited from the Luohu District of Shenzhen City, an eastern city in China, between January and December 2022 for this study. The survey comprised a physical examination and the completion of health-related questionnaires.

## Methods

### Study design and participants

This study adopted a case-control design, conducted from January to December 2022 in Shenzhen, a city in eastern China. A random sample of 80 sanitation workers was recruited from the Luohu District of Shenzhen for the study. They were all male, Han Chinese, and had a mean age of 52.50 (± 5.38) years. It is worth noting that we recruited sanitation workers whose primary job was street sweeping, and we limited the enrolment of study participants who were currently smoking, to better control for confounding factors. The controls consisted of 80 non-sanitation workers recruited during the same period and place. All the controls were male, Han Chinese, with a mean age of 51.91 (± 4.29) years, excluding current smokers. The age distributions of the two groups were not found to be significantly different, with a p-value greater than 0.05 (Table 1). All participants underwent a comprehensive physical examination and completed a series of health-related questionnaires. The physical examination entailed a comprehensive assessment, including laboratory tests, conducted at the Physical Examination Center of Shenzhen Prevention and Treatment Center for Occupational Diseases (SPTCOD). The health-related questionnaires included basic demographic information (age, gender, ethnicity, marital status, and educational attainment) and questionnaires about lifestyle factors (smoking status, alcohol consumption, three basic meals including breakfast, lunch, and dinner, weekly working hours and sleep duration, etc.). Before the commencement of the survey, all participants were required to provide written informed consent. The study was approved by the Ethics Committee of SPTCOD (No. LL2020-34) and by the 1975 Declaration of Helsinki and its later amendments or comparable ethical standards.

**Table 1:** Baseline characteristics of the sanitation worker and control groups.

Variable	Total (n = 160)	Group		*P*
		Controls (n = 80)	sanitation workers (n = 80)	
Age, year	52.21 ± 4.86	51.91 ± 4.29	52.50 ± 5.38	0.447
Sleep duration, hours	6.71 ± 1.29	6.79 ± 1.08	6.63 ± 1.47	0.442
BMI (kg/m^2^)	24.37 ± 2.88	24.74 ± 2.72	23.99 ± 3.00	0.098
Smoking status*, n (%)				**0.023**
never /occasional	124 (77.50)	56 (70.00)	68 (85.00)	
previous	36 (22.50)	24 (30.00)	12 (15.00)	
Physical activity, n (%)				**<0.001**
no	50 (31.25)	14 (17.50)	36 (45.00)	
yes	110 (68.75)	66 (82.50)	44 (55.00)	
Three basic meals, n (%)				**<0.001**
irregularly	86 (53.75)	57 (71.25)	29 (36.25)	
regularly	74 (46.25)	23 (28.75)	51 (63.75)	
Educational attainment, n (%)				**<0.001**
< High school	85 (53.12)	16 (20.00)	69 (86.25)	
≥ High school	75 (46.88)	64 (80.00)	11 (13.75)	
Marital status, n (%)				0.677
Married	154 (96.25)	76 (95.00)	78 (97.50)	
other	6 (3.75)	4 (5.00)	2 (2.50)	
Weekly working hours				0.255
< 48hours	99 (61.88)	46 (57.50)	53 (66.25)	
≥ 48 hours	61 (38.12)	34 (42.50)	27 (33.75)	

Abbreviations: BMI, body mass index; TL, telomere length.

*: The study population did not include any current smokers.

Note: Bolded numbers indicate statistical significance

### Assessments of inflammation indicators

A total of 5.0 mL of upper limb venous blood was collected from each participant and subsequently transferred separately into two tubes: one containing a regular biochemical reagent without anticoagulant and the other containing EDTA-anticoagulant, the EDTA anti-coagulated blood samples were reused for DNA extraction after the complete blood cell count test. We stored the blood samples at -80°C for later DNA extraction and TL measurement after the samples were examined for blood cells and inflammation indicators immediately after collection. The complete blood cell count test was analyzed using Auto Hematology Analyzer (Instrument model: BC-6800Plus manufactured by Shenzhen Mindray Biomedical Electronics Co., Ltd.). Serum albumin concentration was analyzed using Clinical Chemistry analyzers (Instrument model: AU5800 manufactured by Beckman Coulter Co., Ltd.). The IRIs was calculated based on the results of the complete blood cell count (CBC) test. SII (platelet count × neutrophil count/lymphocyte count). Other inflammation-related indices included PLR (platelet count /lymphocyte count), NLR (neutrophil count /lymphocyte count), lymphocyte count (LYM, × 10^−9^/L), and serum albumin concentration (ALB, g/L).

### DNA extraction and TL measurement

The extraction process employs a distinctive buffer system that utilizes silicon-based magnetic beads to specifically adsorb and release nucleic acids, thereby facilitating the separation and purification of nucleic acids. Once the cells had reached approximately 80% confluence, the medium was removed, and the cells were washed twice with phosphate-buffered saline (PBS) solution. 1 mL of DNAzol (Invitrogen) solution was added per 2×10^6^ cells, and after the cells were completely lysed, they were transferred to a centrifuge tube, 0.5 mL of anhydrous ethanol was added, and the tube was gently inverted to mix. The precipitate was washed twice with 75% ethanol and the appropriate amount of TE solution (1 mmol/L EDTA, 10 mmol/L Tris, pH 8.0) was added to dissolve the precipitate. The DNA concentration and purity were determined, the measurement was repeated 3 times for each sample to take the average value, and it was ensured that the optical density D_260_/D_280_ ratio of all samples was between 1.8 and 2.0 and stored at -20°C for spare parts.

Telomere length measurement: The sample DNA was diluted to 17.5 mg/L, 95°C for 10 min, immediately ice bathed for 10 minutes, briefly centrifuged at 730× g, and stored at 4°C protected from light. Each primer (Invitrogen) was diluted to the appropriate concentration (10×) with TE (pH 8.0). Telomeric primer sequences: tel1 GGTTTTGAGGGTGAGG-GTGAGGGTGAGGGTGAGGGTGAGGT, 2.7*μ*mol/L: tel2 TCCCGACTATCCCTATCCCTATCCCTATCCCTATCCCTATCCCTA, 9*μ*mol/L. The single-copy reference gene (β-globin) primer: HBG1 GCTTCTGACACAACTTGTTCACTAGC, 4*μ*mol/L; HBG2 CACCAAC-TTCATCCACGTTCAC, 4*μ*mol/L. Single wells were used with a 20 *μ*L reaction system containing 35 ng of sample genomic DNA, with three replicate wells. All samples were subjected to two sets of amplification: one is telomere fragment (T) amplification, and the other is reference gene (S) amplification. Negative control was set in each group. The reaction was started at 95°C for 10 min. After that, the telomeric repeat fragments were amplified at 95°C, 15s, 54°C, 2 min for 22 cycles, and the single-copy gene was amplified at 95°C, 15 S, 58°C, 1 min for 30 cycles. All reactions were performed using an ABI 7300 Sequence detection system (Applied Biosystems, Foster City CA, USA). Two sets of C_t_ are obtained for each sample, and the difference between the two values with the same hole location is ▲C_t_. The T/S ratio is calculated as T/S ratio = 2 ▲C_t_. Each sample was tested three times, and the results were averaged. The mean value of the T/S ratio for each sample is proportional to its telomere length and is referred to as the mean T/S ratio. For comparison, in an experiment, one sample is selected as a reference and its value is set to 1, and the measurements of the other samples are adjusted accordingly, resulting in the relative T/S ratio.

#### Covariates assessment

The assessed covariates included age (year), body mass index (BMI, kg/m^2^), sleep duration (hour), smoking status (never or occasional/previous), physical activity (no/yes), three basic meals (regularly / irregularly), educational attainment (<High school / ≥High school), marital status (married / others), weekly working hours (<48 hours / ≥48 hours). It is worth noting that the participants in this study were all ex-smokers or never smokers. None were current smokers.

### Statistical analysis

The assessment was conducted to compare baseline conditions, and differences in inflammation-related markers and TL, and to investigate the relationship between inflammation-related markers and TL between sanitation workers and controls. Categorical variables were identified as percentages [n (%)]. If the variables in question displayed a normal distribution, they were expressed as a mean and standard deviation (mean ± SD). For variables exhibiting non-normal distributions, the median and quartiles (M ± IQR) were employed for expression. To ascertain the baseline characteristics of the two groups, student t-tests were employed in the case of continuous variables, while chi-square tests were used concerning categorical variables. Multivariate linear regression was used to calculate regression coefficients (β) and 95% confidence intervals (CIs) of IRIs on TL for the sanitation worker and control groups, respectively. Model 1 accounted for age and BMI. Model 2 further accounted for additional variables, including sleep duration, smoking status, physical activity, three basic meals, educational attainment, marital status, and weekly working hours. Furthermore, a restricted triple spline was employed to investigate the dose-response relationship between IRIs and TL in the Sanitation workers group and control groups. Statistical analyses were performed using R 4.3.1 [29], and a significance level of <0.05 (two-tailed) was considered statistically significant.

## Results

**Fig 1:**
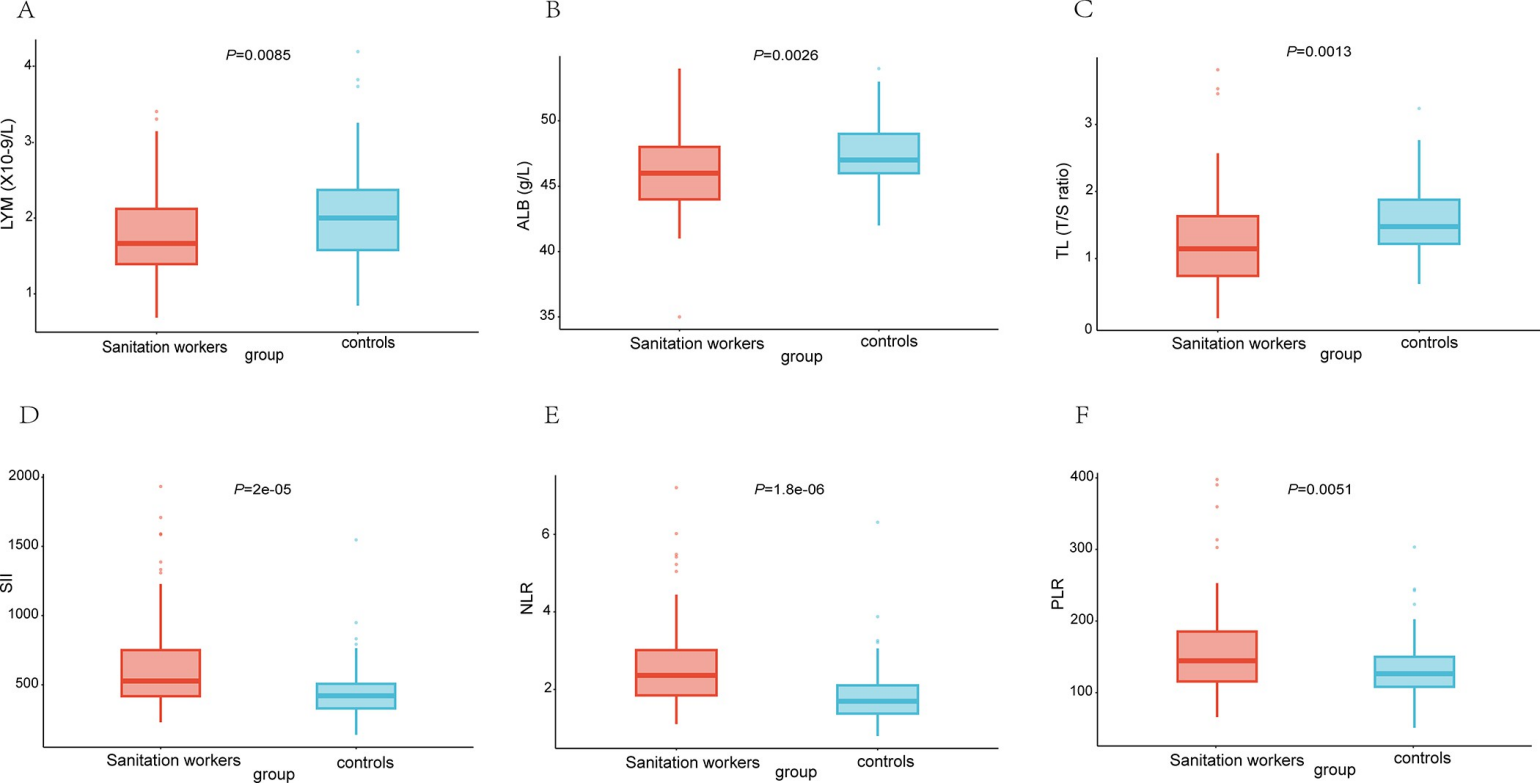
Comparison of inflammation-related markers and telomere length between sanitation worker and control groups. Abbreviations: ALB, serum albumin concentration; LYM, lymphocyte count; SII, Systemic Inflammation Index; PLR, Platelet to lymphocyte ratio; NLR, Neutrophil to lymphocyte ratio. (A): The level of LYM was found to be lower in the sanitation worker group compared to the control group. (B): The mean ALB was found to be lower in the sanitation worker group compared to the control group. (C): Compared to the control, the telomere length was lower in the sanitation worker group. (D): The SII was found to be significantly higher in the sanitation worker group than in the control group. (E): The NLR was found to be significantly higher in the sanitation worker group than in the control group. (F): The PLR was found to be significantly higher in the sanitation worker group than in the control group.

Table 1 presents the baseline for the Sanitation Workers and the controls. In comparison to the controls, the sanitation workers exhibited lower levels of education, more irregular meal patterns, and lower rates of regular exercise. The two groups did not differ statistically regarding age, BMI, sleep duration, or marital status. Moreover, the sanitation workers exhibited shorter TL, lower LYM, and ALB, but higher SII, PLR, and NLR (Fig 1).

The one-factor linear regression analysis did not demonstrate statistically significant correlations between telomere length and factors such as age, BMI, sleep duration, smoking status, physical activity, three basic meals, educational attainment, marital status, weekly working hours, ALB, SII, PLR, and NLR in either the sanitation workers or the control group. However, a negative relation was observed between LYM and TL in the group of sanitation workers. (β = -0.31, 95% CI: -0.57, -0.05) (Table 2).

**Table 2:** Linear regression with one variable analysis between various factors on telomere length in sanitation workers and control groups.

Variables	Sanitation workers		Controls	
	β (95%CI)	*P*	β (95%CI)	*P*
Age (year)	-0.00 (-0.04, 0.03)	0.768	-0.02 (-0.04, 0.01)	0.***235***
BMI (kg/m^2^)	0.01 (-0.05, 0.06)	0.756	-0.01 (-0.05, 0.04)	0.765
Sleep duration (hours)	-0.08 (-0.19, 0.03)	0.183	0.02 (-0.09, 0.13)	0.725
LYM (×10^−9^/L)	**-0.31 (-0.57, -0.05)**	**0.024**	-0.06 (-0.24, 0.12)	0.531
ALB (g/L)	0.02 (-0.04, 0.07)	0.542	0.01 (-0.04, 0.06)	0.636
SII	0.00 (-0.00, 0.00)	0.734	0.00 (-0.00, 0.00)	0.332
PLR	0.00 (-0.00, 0.00)	0.505	0.00 (-0.00, 0.00)	0.299
NLR	0.09 (-0.05, 0.23)	0.204	0.08 (-0.07, 0.23)	0.324
Smoking status				
never /occasional	0.00 (Reference)		0.00 (Reference)	
previous	-0.21 (-0.67, 0.25)	0.37	0.17 (-0.08, 0.42)	0.181
Physical activity				
no	0.00 (Reference)		0.00 (Reference)	
yes	0.03 (-0.30, 0.36)	0.867	-0.02 (-0.32, 0.29)	0.909
three basic meals				
irregularly	0.00 (Reference)		0.00 (Reference)	
regularly	0.16 (-0.18, 0.50)	0.373	0.06 (-0.20, 0.31)	0.665
Educational attainment				
< High school	0.00 (Reference)		0.00 (Reference)	
≥ High school	0.03 (-0.44, 0.51)	0.893	-0.14 (-0.43, 0.15)	0.335
Marital status				
married	0.00 (Reference)		0.00 (Reference)	
others	-0.36 (-1.41, 0.69)	0.499	-0.22 (-0.75, 0.31)	0.418
Weekly working hours				
< 48hours	0.00 (Reference)		0.00 (Reference)	
≥ 48 hours	0.06 (-0.29, 0.41)	0.729	0.16 (-0.07, 0.40)	0.169

Abbreviations: BMI, body mass index; ALB, serum albumin concentration; LYM, lymphocyte count; SII, Systemic Inflammation Index; PLR, Platelet to lymphocyte ratio; NLR, Neutrophil to lymphocyte ratio.

Note: Bolded numbers indicate statistical significance

Table 3 provides the findings of the multivariate linear regression model. In Model 1, after accounting for age and BMI, there was a negative correlation between LYM and TL in the sanitation workers (β = -0.38, 95% CI: -0.66, -0.10), while the correlation was not statistically significant in the controls. Further adjustment for other potential confounding variables in Model 2 revealed that LYM remained correlated with TL in sanitation workers (β = -0.36, 95% CI: -0.65, -0.07). However, the correlations of ALB, SII, PLR, and NLR with TLwere not significant in either group.

**Table 3:** Multivariate linear regression analysis of inflammatory markers and telomere length in sanitation worker and control groups.

	Sanitation workers		Controls	
	Model 1 β (95% CI)	Model 2 β (95% CI)	Model 1 β (95% CI)	Model 2 β (95% CI)
LYM (×10-9/L)	**-0.38 (-0.66, -0.10)**	**-0.36 (-0.65, -0.07)**	-0.05 (-0.24, 0.13)	-0.06 (-0.26, 0.14)
*P*	**0.010**	**0.019**	0.795	0.528
ALB (g/L)	0.02 (-0.04, 0.08)	0.03 (-0.03, 0.09)	0.01 (-0.04, 0.06)	0.01 (-0.04, 0.06)
*P*	0.551	0.388	0.568	0.728
SII	0.00 (-0.00, 0.00)	0.00 (-0.00, 0.00)	0.00 (-0.00, 0.00)	0.00 (-0.00, 0.00)
*P*	0.670	0.597	0.282	0.183
PLR	0.00 (-0.00, 0.00)	0.00 (-0.00, 0.00)	0.00 (-0.00, 0.00)	0.00 (-0.00, 0.01)
*P*	0.406	0.311	0.317	0.146
NLR	0.11 (-0.04, 0.25)	0.10 (-0.05, 0.25)	0.09 (-0.06, 0.24)	0.09 (-0.07, 0.25)
*P*	0.157	0.210	0.258	0.277

Model 1: Adjustment for age (year), BMI (kg/m^2^).

Model 2: Adjustment for age (year), BMI (kg/m^2^), sleep duration (hour), smoking status (never or occasional /previous), physical activity(no/yes), three basic meals(regularly/irregularly), educational attainment (< high school/≥high school), marital status (married/ others), weekly working hours (<48hours / ≥48 hours).

Note: Bolded numbers indicate statistical significance

**Fig 2:**
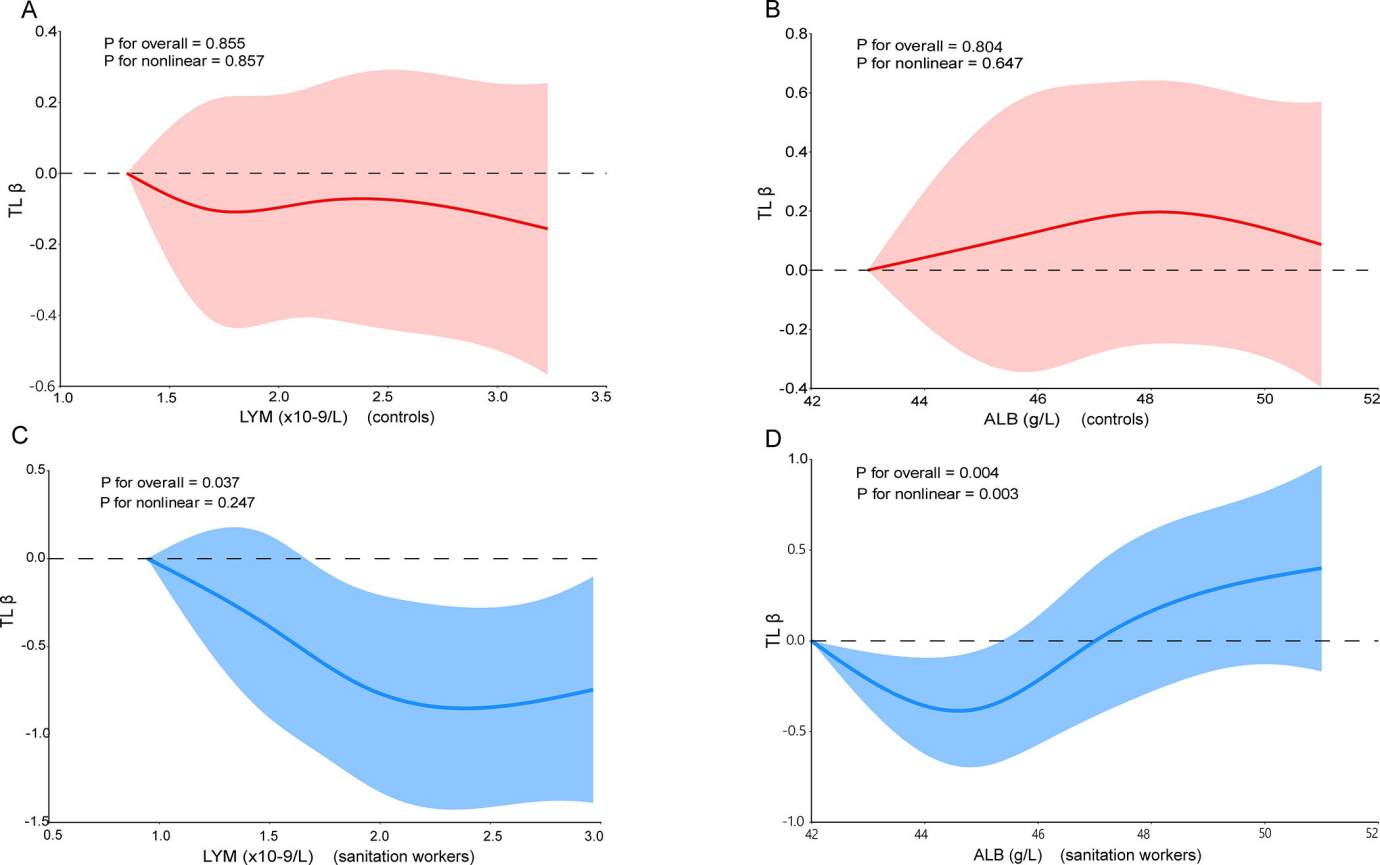
The relationship between serum albumin concentration, lymphocyte count, and telomere length in sanitation worker and control groups was fitted using restricted cubic spline curves. Adjustment for age(year), BMI (kg/m^2^), sleep duration (hour), smoking status (never or occasional /previous), physical activity (no/yes), three basic meals (regularly/irregularly), educational attainment (< high school/≥high school), marital status (married/ others), weekly working hours (<48hours/≥48 hours). (A): Non-linear relationship between LYM and TL in the control group. (B): Non-linear relationship between ALB and TL in the control group. (C): Non-linear relationship between LYM and TL in the sanitation worker group. (D): Non-linear relationship between ALB and TL in the sanitation worker group.

A restricted cubic spline was employed to analyze the dose-response relationship between IRIs and TL. After adjusting for potential confounding variables, we observed a non-linear relationship between ALB and TL in sanitation workers (*P* for nonlinearity = 0.004) (Fig 2).

## Discussion

We found that novel inflammatory markers (SII, PLR, and NLR) exhibited significantly elevated levels in the sanitation workers in comparison to the controls. The LYM, ALB, and TL levels were lower in the sanitation workers compared to the controls. Furthermore, a negative relation was observed between LYM and TL in the sanitation workers, whereas no such correlation was evident in the controls. What is more, a non-linear relationship was identified between ALB and TL in sanitation workers.

The findings of our study indicate that a reduction in LYM is positively related to TL shortening in sanitation workers. Our findings are supported by a recent study in which TL was associated with lymphocyte count in elderly patients with coronavirus disease in 2019 [30]. Additionally, several studies have demonstrated a correlation between the proliferative capacity of T lymphocytes and TL [31, 32]. Furthermore, we observed that novel inflammatory markers (SII, NLR, and PLR) were elevated in sanitation workers compared to the control population (general population). A study by Colella *et al*. demonstrated that patients with sickle cell disease exhibited shorter telomeres, which may be directly related to inflammatory markers [33]. It can be reasonably concluded from previous research findings that inflammation is an important factor contributing to telomere shortening [20]. Indeed, low LYM is a manifestation of inflammation [22]. Furthermore, a significant number of investigations have demonstrated a correlation between LYM and age-related diseases [23–25, 34]. Our results provide further evidence that inflammation could be an important mechanism for telomere shortening.

We found that ALB was lower in the sanitation workers compared with the controls. Low ALB concentration was reported to be associated with inflammation [35, 36]. This may be related to the low economic and social status of the sanitation workers and low-quality lifestyle. *etc*. In addition, several studies have reported that low ALB levels are linked to a greater risk of mortality over an extended period among elderly individuals [37–39]. Decreased ALB is strongly associated with aging, reflecting conditions of inflammation, frailty, and a variety of pathologies, including cancer, deforming arthritis, and hepatic malfunction [26, 27]. Marjolein *et al*. indicated that low ALB may be associated with an increased risk of developing sarcopenia [28]. These studies support our findings that lower ALB in sanitation workers is associated with shortened TL.

### Strengths and limitations

In the authors’ knowledge, this represents the inaugural study to investigate the correlation between IRIs and TL in sanitation workers. Nevertheless, the assessment of the findings is constrained by the following factors. First, the cross-sectional design precludes the drawing of causal inferences. Although we adjusted for several important confounders, unidentified or residual confounders may still cause bias. For example, we did not measure factors such as the level of dust exposure of sanitation workers and the economic and social status of the study participants. Further cohort or intervention studies are also needed to confirm our findings.

## Conclusion

The results of the study demonstrated a correlation between LYM and ALB with shortened TL in sanitation workers, and the novel inflammatory markers (SII, PLR, and NLR) were higher in sanitation workers than the controls. This study provides new evidence for the effect of elevated inflammation on accelerated aging in Chinese sanitation workers. We recommend that local governments and relevant authorities take steps to reinforce the protection of occupational health and labor rights for sanitation workers. This would serve to enhance their occupational status and health.

## Supporting information

S1 Table Sanitation worker data. (XLSX)

S2 Table Control group data. (XLSX)
